# Special Issue “Advances in the Physiology of Primary and Secondary Plant Metabolism Under Abiotic and Biotic Stress”

**DOI:** 10.3390/ijms252212339

**Published:** 2024-11-17

**Authors:** Natalia Zagoskina

**Affiliations:** K.A. Timiryazev Institute of Plant Physiology, Russian Academy of Sciences, 127276 Moscow, Russia; nzagoskina@mail.ru

One of the most relevant areas of biology is the study of plant adaptation processes to the action of various stress factors of abiotic and biotic nature, which is reflected in the works of molecular biologists, geneticists, microbiologists, plant physiologists, and biochemists, as well as biotechnologists [1,2,3,4,5,6,7,8,9,10]. Considerable attention is paid to assessing the effects of temperature conditions (especially high temperatures), light exposure (intensity, duration, qualitative composition), UV radiation, drought, ozone, heavy metals, soil salinization, pathogens, and other influences [11,12,13,14,15,16,17,18]. All of them have a significant and often negative impact on the ecology, including the vital activity and productivity of plants. In the case of agricultural crops of industrial use, this is accompanied by significant losses in their productivity and the quality of the substances obtained. We must not forget that plants make a significant contribution to the preservation of a comfortable environment for the vital activity of various organisms and are also producers of both primary (amino acids, carbohydrates, lipids, etc.) and secondary (polyphenols, terpenoids, alkaloids, etc.) metabolites for food and medicinal use [19,20,21,22,23,24,25,26,27]. All these compounds are important and necessary for the health of the world’s population, which requires studying the peculiarities of their formation, including under stress conditions [28,29,30,31,32].

This Special Issue presents two reviews and eleven original research papers with great scientific potential. Each article reflects new and original innovative approaches to assessing the effects of various stress factors on plants, demonstrating the development of this scientific field through the successful use of not only classical physiological, biochemical, and genetic methods but also the most modern molecular biological and metabolomic approaches.

Oxygen is important and necessary for the vital activity of plants [33,34,35,36]. Only under certain conditions can they exist in conditions of its deficiency or even complete absence [37,38,39,40]. In a review by Yemelyanov et al. [41] various aspects of plant adaptation to oxygen deficiency are considered, paying great attention to their metabolomics. The latest achievements in the field of the specificity of plant metabolic profiles during the lack of oxygen, native hypoxia, and reoxygenation are presented. The issues of oxidative stress metabolism are considered. It is noted that in the study of metabolites and/or metabolic pathways specific to the processes of hypoxia and/or anoxia, in addition to the already traditional metabolic subspecies, a discipline such as fluxomics should be distinguished, which allows quantifying metabolic flows.

The unique secondary metabolites of plants synthesized in all plant cells include various polyphenols [42,43,44,45]. Their functional role is associated with the processes of photosynthesis, respiration, hormonal regulation, resistance, and stress adaptation [46]. They are extremely diverse in chemical properties and biological activity, and their composition and content depend on the species of plants and the growing and environmental conditions. The presented review provides information on various classes of polyphenols, their structure, properties, antioxidant activity, and biosynthesis, including molecular genetic aspects (genes, transfactors, etc.), the effect of various abiotic factors (light, UV radiation, temperature, heavy metals) on their accumulation and composition, as well as metabolic regulation. Information is provided on plant polyphenols as important and necessary components of functional nutrition and pharmaceutically valuable substances for public health. Proposals on promising areas of research and development in the field of plant polyphenols are presented.

One of the important areas of biological research is the study of the plants’ adaptation mechanisms to stress, as well as the search for factors and/or regulators that contribute to reducing their negative effects [47,48,49,50]. In this case, not only classical physiological and biochemical research methods are used, but also molecular genetics and metabolomic methods [51,52,53,54]. Various abiotic and biotic factors, as well as substances synthesized directly in plant cells, can act as regulators of stress reactions [55,56,57,58,59,60]. These include extracellular polysaccharides (EPSs) isolated from the submerged cultures of twenty basidiomycete strains assigned to thirteen species and eight genera [61]. The treatment of wheat seedlings with EPS preparations improved their growth, increased the activity of antioxidant enzymes (peroxidase, superoxide dismutase), and influenced the level of lipid peroxidation and accumulation of polyphenols. All this indicates the phytostimulating activity of EPSs (extracellular metabolites produced in the xylotrophic basidiomycete cultures) and the possibility of their successful use as stress-protective compounds.

Original data are presented on the role of anthraquinone parietin in maintaining the viability of lichen *Xanthoria parietina* during dehydration [62]. In its absence, there was an increase in lipid peroxidation and a decrease in membrane stability during thalli drying, which is not typical for its accumulation. Highly pigmented lichens had thicker cell walls and higher water retention capacity relative to thalli with low pigmentation levels. All this indicates the important role of such a secondary metabolic compound as parietin in the resistance of *X. parietina* to dehydration.

Essential oils (Eos), which are successfully used in medicine, food, cosmetics, the perfume industry, and agriculture, are of great interest [63,64,65,66,67]. The regulation of their accumulation in plants, including under stressful conditions (different humidity levels, elevated temperatures), was studied in *Anethum graveolens* plants grown under control conditions or treated with cytokinin (6-benzylaminopurine) [68]. A change in the morphology of leaf plates, the inhibition of flowering, an increase in the content of Eos and its main components α-fellandren and p-cymen in leaves, and limonene in the umbrellas and fruits of plants were noted. At the same time, the increased accumulation of Eos in dill leaves was longer with sufficient moisture. On the contrary, under conditions of heat and water deficiency, the effect of cytokinin treatment on the accumulation of Eos in leaves was short-lived and did not manifest itself on umbellets and fruits. All this indicates a change in the adaptive properties of *A. graveolens* after treatment, prolonging the “youth” of vegetative organs and the ability to biosynthesize Eos under conditions of sufficient moisture.

Chitosan is one of the exogenous bioelicitors that protect plants from stress [69,70,71,72]. To a large extent, its effectiveness is associated with the activation of the secondary metabolites’ accumulation, including phenolic compounds. On plants *Salvia abrotanoides* Karel and *S. yangii*. The effects of chitosan and water stress were studied by HPLC and transcriptomics to predict the patterns of phenolic flavonoids and the accumulation of terpenoid components [73]. It was shown that the use of bioelicitor on leaves at doses of 100 and 200 mg/L under conditions of good watering and moderate stress increased the content of hydroxyl cryptotanshinone (OH-CT) and cryptotanshinone (CT) as the main terpenoid components in both species. Under these conditions, there were changes in the activity of some genes in their biosynthesis (DXS2, HMGR, KSL). The thesis is put forward that the action of chitosan can lead to various effects in the adaptation of plants to a stressor, depending on the method of processing and concentration, as well as the conditions of their growth and species specificity.

Another actively developing area in the field of physiology and biochemistry of stress reactions of plants is the assessment of their adaptation to the heavy metals’ action [17,54,74,75,76]. These studies are especially important in connection with significant changes in the ecology of the environment, which requires the development of systems for its protection [77,78]. Using *Nicotiana tabacum* plants, the long-term impact of copper sulfate on their physiological state, copper content, activity of antioxidant enzymes, accumulation of polyphenols, and tissue lignification during 40 days of cultivation was studied [79]. Changes in metabolic activity under the stress of this metal were more pronounced in the axial organs of plants (roots, stems) and were associated with the composition and amount of phenolic compounds and lignin. The increases in the amounts of ferulic, cinnamic, and *p*-coumaric acids in root tissues in response to an excess of copper can be considered markers of the lignification process [80]. Analysis of the individual components of the phenolic complex and their distribution in the axial organs of *N. tabacum* indicate that prolonged exposure to copper led to specific changes in the spectrum of phenolic compounds and the amount of lignin.

The study of the separate and complex effects of various concentrations of aluminum (0 mM, 0.4 mM, and 1 mM) and calcium (0.8 mM and 5.6 mM) was carried out on tea plants (*Camellia sinensis*), evaluating root growth, amino acid and phenolic compound content, and gene expression [81]. According to the data obtained, calcium had a stressful effect on plants, inhibited root growth, and led to metabolic disorders, mainly reducing the content of most amino acids and organic acids in roots, stems, and leaves. According to literature data, the reaction of plants to the action of calcium depends on its concentration, duration of exposure, type of crop, and growing conditions [82,83]. Under the action of aluminum, root growth increased, as did the accumulation of valuable metabolites of a phenolic nature, and this effect depended on the concentration of the metal. In general, calcium stress caused severe growth inhibition and metabolic disorders in *C. sinensis*, which could have been prevented by the action of aluminum, especially with regard to the preservation of root tips and the accumulation of secondary metabolites.

The regulation of plant metabolism is important for maintaining the viability of plants under the influence of biotic stress factors, which has been studied by the example of the action of gall-forming insects (*Caillardia azurea*, *Asiodiplosis noxia*, *Caillardia robusta*, *Aceria haloxylonis*) on *Haloxylon aphyllum* and *H. persicum*. [84]. Under conditions of biotic stress, changes in almost all anatomical structures of shoots were noted, which were less pronounced in *H. persicum* than in *H. aphyllum*. At the same time, the biosynthesis of fatty acids and γ-tocopherol was activated in *H. aphyllum*, whereas *H. persicum* also contains dialkyl esters, carbohydrates and their derivatives, aromatic acids, phytosterols, polyphenols, and terpenoids. All this indicates a more pronounced modulation of metabolic pathways in *H. persicum*, which plays a crucial role in increasing its survival and growth under biotic stress. The need for research into the interaction of insects and plants, as well as the study of the impact of gall formers on their metabolism and resistance, has been reported in the literature [85,86].

Of particular interest is the work performed on *Nicotiana tabacum* transgenic plants overexpressing the choline oxidase gene from *Arthrobacter globiformis* to assess the effect of salt stress [87]. The biochemical, cytological, and molecular biological characteristics of transgenic and wild-type (WT) plant leaves were determined. An important effect was the correction of the rate of transgenic codA line development under salt exposure, the restoration of the plastid structural organization, accompanied by an increase in the amount of chlorophylls, and the regulation of concomitant systems of primary and secondary metabolism. All these data once again confirm the value of using transgenic plants as model systems for studying adaptation to stress [88,89].

Another area of research presented in this Special Issue is the study of the morphometric characteristics and genetic variability of the ISSR marker in *Rhodiola rosea* from various ecological and geographical zones of the Altai Republic. The plants were harvested in undisturbed territories as well as after grazing, that is, under conditions of anthropogenic impact [90]. The variability of morphometric characteristics of living pubescent female plants of the *R. rosea* was assessed, and a comparative analysis of genetic variability in cenopopulations was carried out. Based on the data obtained, the authors conclude that it is necessary to protect the *R. rosea* gene pool in the Altai Republic under anthropogenic stress. The impact of anthropogenic factors on various plant communities is one of the environmental problems, and the study of these issues has important fundamental and practical significance [91,92]. The material on the temporal and geographical dynamics of potato virus Y (PVY) diversity in Russia is also original [93]. This viral pathogen of potato has several genetic variants and geographic distributions that can be influenced by environmental factors, aphid vectors, and reservoir plants [94,95]. The PVY populations transmitted by aphids to potato plants in various climatic zones of Russia, namely the Moscow and Astrakhan regions, were studied. At the same time, a significantly greater diversity of PVY isolates was found in the Astrakhan region, where winters are shorter and milder and summers are warmer compared to the Moscow region. Data on their specific types are provided. All these recombinants were composed of the genome sections derived from PVY types O and N, but no full-length sequences of such types were present.

In conclusion, I would like to express my sincere gratitude to the authors for their interest and trust during the submission, review, and acceptance of articles for publication. The contribution of the administrative staff, editors, and all other employees of the *International Journal of Molecular Sciences* is also important; their prompt and responsible work, as well as assistance in resolving various issues, contributed to the preparation of this Special Issue. Thanks to this common work, we can gain new knowledge in this interesting and exciting area of research, as well as come closer to understanding the mechanisms of plant adaptation to stress effects of various natures.
